# DNA Methylation and RNA-Sequencing Analysis Show Epigenetic Function During Grain Filling in Foxtail Millet (*Setaria italica* L.)

**DOI:** 10.3389/fpls.2021.741415

**Published:** 2021-08-27

**Authors:** Tao Wang, Quanwei Lu, Hui Song, Nan Hu, Yangyang Wei, Pengtao Li, Yuling Liu, Zilin Zhao, Jinrong Liu, Baohong Zhang, Renhai Peng

**Affiliations:** ^1^College of Biology and Food Engineering, Anyang Institute of Technology, Anyang, China; ^2^Innovation and Practice Base for Postdoctors, Anyang Institute of Technology, Anyang, China; ^3^Anyang Academy of Agriculture Sciences, Anyang, China; ^4^Department of Biology, East Carolina University, Greenville, NC, United States

**Keywords:** DNA methylation, grain filling, gene expression, foxtail millet, crop

## Abstract

Grain filling is a crucial process for crop yield and quality. Certain studies already gained insight into the molecular mechanism of grain filling. However, it is unclear whether epigenetic modifications are associated with grain filling in foxtail millet. Global DNA methylation and transcriptome analysis were conducted in foxtail millet spikelets during different stages to interpret the epigenetic effects of the grain filling process. The study employed the whole-genome bisulfite deep sequencing and advanced bioinformatics to sequence and identify all DNA methylation during foxtail millet grain filling; the DNA methylation-mediated gene expression profiles and their involved gene network and biological pathway were systematically studied. One context of DNA methylation, namely, CHH methylation, was accounted for the largest percentage, and it was gradually increased during grain filling. Among all developmental stages, the methylation levels were lowest at T2, followed by T4, which mainly occurred in CHG. The distribution of differentially methylated regions (DMR) was varied in the different genetic regions for three contexts. In addition, gene expression was negatively associated with DNA methylation. Evaluation of the interconnection of the DNA methylome and transcriptome identified some stage-specific differentially expressed genes associated with the DMR at different stages compared with the T1 developmental stage, indicating the potential function of epigenetics on the expression regulation of genes related to the specific pathway at different stages of grain development. The results demonstrated that the dynamic change of DNA methylation plays a crucial function in gene regulation, revealing the potential function of epigenetics in grain development in foxtail millet.

## Introduction

Gene expression is not only controlled by DNA sequences but also by epigenetic marks in eukaryotes. DNA methylation as one of the important epigenetic modifications has been demonstrated as closely related to gene expression in biological processes, such as transcriptional activity, developmental regulation, and environmental responses (Maunakea et al., 2010; Huang et al., 2019; Xing et al., 2020; Zhu et al., 2020). In eukaryotes, the methylation at the 5′ position of cytosine (5 mC) is the main type of DNA methylation, which could be found to occur in the CG, CHG contexts (symmetrical), or in CHH contexts (asymmetrical) in various regions of a genome (Huang et al., 2019). Many emerging studies proved that methylated DNA is widely involved in plant growth regulation. The level of DNA methylation decreased gradually during the development of tomato and strawberry fruits, and the application of methyltransferase inhibitor promoted premature ripening of tomato and strawberry fruits (Zhong et al., 2013; Cheng et al., 2018). Changes in DNA methylation were also observed during pepper ripening (Xiao et al., 2020). In contrast, the orange fruit showed obvious DNA hypermethylation as ripening (Huang et al., 2019). However, the function and dynamic change of methylated DNA have not been reported during grain filling in foxtail millet.

Grain filling could affect grain quality and yield in many crops as it is the most important developmental phase (Saini and Westgate, 1999). The grain filling process is complicated and can be regulated by endogenous metabolism and the external environment. Sucrose and starch metabolism are closely related to grain filling (Kato et al., 2007). Increasing expression of SuSase genes enhance the breakdown of sucrose in spikelets and promoted the transport of sucrose to grains (Ranwala and Miller, 1998). Grain filling could be improved through promoting starch synthesis done by *GIF2*, which also encodes an ADP-glucose pyrophosphorylase large subunit. Inhibition of *GIF2* had a negative effect on grain filling rate and yield (Wei et al., 2017). The grain size in rice was regulated by *GSA1* through encoding a UDP-glucosyltransferase (Dong et al., 2020). Plant hormones could also affect spikelet development and participate in regulating grain filling. Grain development could be prolonged through auxin and cytokinin done by DEP1/qPE9-1, which also promotes starch accumulation (Zhang et al., 2019). Abscisic acid (ABA) biosynthesis also promoted endosperm cell division and grain-filling (Yang et al., 2006; Wang et al., 2015). Polyamines (PAs) which regulated various metabolic processes in plants could also regulate grain filling (Chen et al., 2013; Liu et al., 2016). High-throughput sequencing technology makes it possible to identify genes associated with grain filling. Gene expression dynamics during grain development have been studied through transcriptome sequencing in different plant species (Wang et al., 2019, 2020b; Wen et al., 2019). The previous study found that genes, related to polyamine metabolism, sucrose-starch conversion, and hormone metabolism, were specifically expressed during grain development which indicates that they may play specific roles at different stages of grain filling in foxtail millet (Wang et al., 2020).

Foxtail millet is an ancient crop originating in China which is a C_4_ cereal with many elite traits, including high tolerance to drought stresses (Lata et al., 2013). Additionally, the suitable genome size, low repetitive DNA, and short life cycle of foxtail millet make it an ideal model plant for studying C_4_ photosynthesis and abiotic stress tolerance (Muthamilarasan and Prasad, 2015; Peng and Zhang, 2021). However, the study on molecular mechanisms of important growth and development processes in foxtail millet is limited compared with rice, maize, and wheat. Especially, the epigenetic modification and the potential function of DNA methylation are limited during grain filling in foxtail millet.

The objective of this study is to explore the potential connection between epigenetic modification and transcription changes during grain development. To achieve this, the study employed the whole-genome bisulfite sequencing (BS) approach to probe the epigenetic changes associated with grain filling at five different development stages in foxtail millet. These results are helpful for us to understand the effect of epigenetics in grain filling in foxtail millet and potentially other C_4_ cereals.

## Materials and Methods

### Plant Materials, Whole-Genome BS Library Construction, and Sequencing

The seeds of foxtail millet variety “Yu Gu 18” were planted and grown in the experimental station of Anyang Institute of Technology (Anyang, Henan, China). The same-aged panicle with the same size was harvested from T1–T5 developmental stages (T1: 7 days after anthesis, T2: 14 days after anthesis, T3: 21 days after anthesis, T4: 28 days after anthesis, and T5: 35 days after anthesis). The experimental field, agronomical practices situation, and sampling process are described in the previous study (Wang et al., 2020).

In isolating genomic DNA, DNeasy Blood and Tissue Kit (Qiagen, Dusseldorf, Germany) was used. Fifteen high-quality BS libraries were constructed and then sequenced with the Illumina Hiseq X Ten platform by Shanghai OE Biotech (Shanghai, China). Briefly, DNA was first fragmented to an approximate size of 250 bp. Then, dA was added to the 3′ end by blunt-end cloning and methylated adaptor ligation. The EZ DNA Methylation-Gold kit (ZYMO, Tustin, CA, United States) was used to bisulfite-convert the ligated DNA. Different DNA fragment lengths were excised from a 2% agarose gel and purified and amplified by PCR.

### BS-Seq Data Analysis

All sequence data were deposited into the NCBI database (Accession number: PRJNA699635). Clean data were obtained by filtering the low-quality sequences and mapped to the foxtail millet genome (Bennetzen et al., 2012) using the mapping program, Bismark (Krueger and Andrews, 2011).

Differentially methylated regions (DMRs) were identified by MethylKit (Akalin et al., 2012), which used a sliding window approach. The window was 1,000 bp, and the step length was 100 bp. The logistic regression was applied to detect significant DMRs and the screened criteria as methylation difference of ≥10% and *p* ≤ 0.05. After DMRs were identified, differentially methylated genes (DMGs) located in DMRs were characterized.

For the identification of differentially methylated promoter (DMP), 2 kb upstream of the transcription start site (TSS) was used as the gene promoter region, and the methylation level in this region was counted. The methylation level of the promoter region between different samples was compared. To analyze DMP between groups, a *t*-test was used. The differences in methylation levels of all genes among all groups were analyzed. DMP regions were screened according to methylation differences of ≥10% and *p* ≤ 0.05.

### RNA Extraction, Library Construction, and Sequencing

The total RNA of kernels from spikelets was extracted using a mirVana miRNA Isolation Kit (Ambion, Inc., Austin, TX, United States) with three biological replicates. Afterward, high-quality libraries were sequenced on the Illumina HiSeq 2500 platform (Illumina, San Diego, CA, United States) with 125 paired-end sequencings. The data analysis was performed according to previously described methods (Wang et al., 2020).

### Integration of DNA Methylation and Gene Expression Analysis

For integration with DNA methylation, RNA-seq data were used representing the same stages of grain filling in foxtail millet from the previous study.

The Pearson correlation analysis of RNA-seq data and DNA methylation levels in each sample was completed as previously described (Jin et al., 2014).

### Gene Function Analysis and Enrichment

Differentially expressed genes associated with DMR were subjected to Kyoto Encyclopedia of Genes and Genomes (KEGG) analysis^1^ (Kanehisa et al., 2008) to map them to the specific pathways they were involved in using the Blast_v2.2.26 software.

## Results

### BS of Foxtail Millet at Different Stages During Grain Filling

**FIGURE 1:**
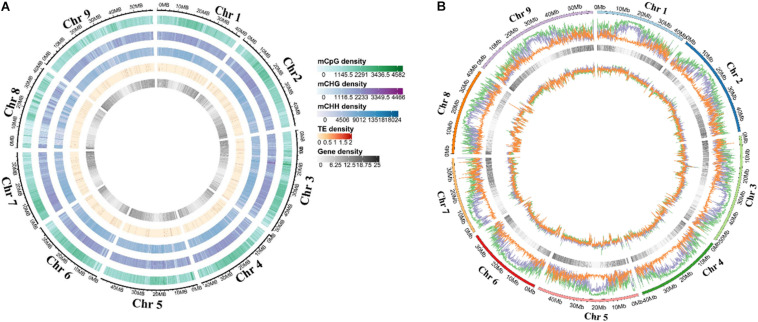
Distribution of DNA methylation in each foxtail millet chromosome. **(A)** Circos plots of foxtail millet chromosomes. Track order: density plot of 5 mC in CG, CHG, and CHH contexts; density of TEs; gene density of each chromosome at the T1. **(B)** Density plot of 5 mC in CG (green), CHG (purple), and CHH (orange) contexts in the gene bodies on each chromosome at the T1.

Deoxyribonucleic acid libraries for five different stages were constructed for sequencing to get the characteristics and change of DNA methylation during grain development. A total of 2.16 billion raw reads were obtained, and the mapping rate was 76.6–83.5%. Moreover, conversion rates of each library were >99.56% (Supplementary Table 1). Sequencing depth was >30-fold coverage per DNA strand (Supplementary Table 2). Furthermore, the proportion of the 5 mC methylation for three contexts in each chromosome was shown in Supplementary Figure 1. The genome-wide pattern of methylated DNA was similar across chromosomes at different stages (Figure 1A and Supplementary Figure 2). The methylation level of different chromosomal positions showed significant differences, and the lowest CHH methylation was found across the genome (Figure 1B and Supplementary Figure 3).

### DNA Methylation Dynamics During Grain Filling in Foxtail Millet

**FIGURE 2:**
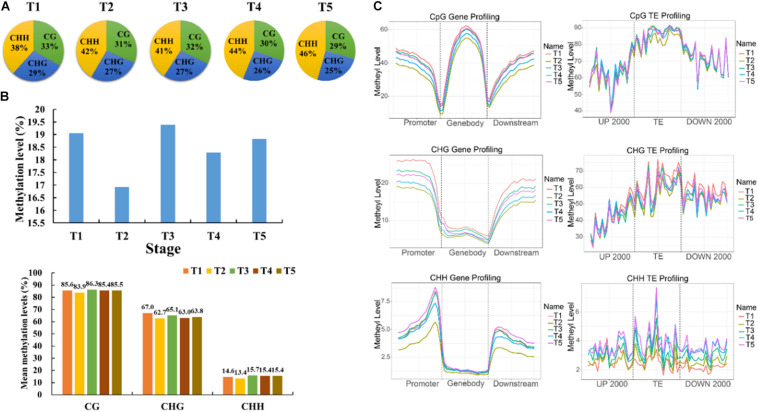
Characteristics of DNA methylation in foxtail millet. **(A)** Relative proportions of mCs in three sequence contexts (CG, CHG, and CHH) in foxtail millet. **(B)** mC, mCG, mCHG, and mCHH methylation levels from 7 to 35 DAA. **(C)** Methylation levels (%) of CG, CHG, and CHH among gene/TE bodies and their 2-kb upstream and downstream regions at different stages.

**FIGURE 3:**
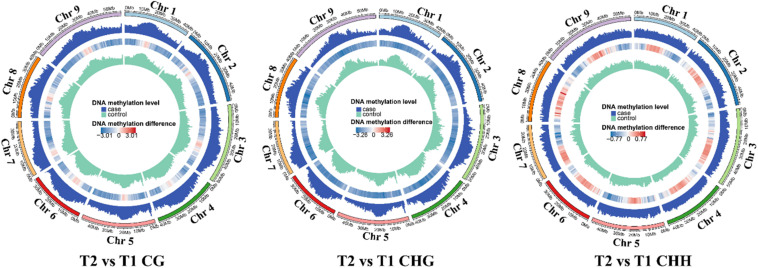
Comparative analysis of DNA methylation levels in different genomic regions.

The context CHH methylation was accounted for the largest percentage during the whole grain filling stage followed by CG and CHG methylation. Furthermore, CHH methylation remained elevated during grain filling (38–46%), but CG and CHG methylation showed the opposite trend (33–29 and 29–25%, respectively) (Figure 2A). The mean methylation levels were lowest at T2, followed by T4, which mainly occurred in the CHG context (Figure 2B). Moreover, methylation level in the promoter, gene-body, and downstream regions of genes also reached the bottom at T2, and then at T4 (Figure 2C). Besides, methylation patterns of TEs were compared among different stages. No significant differences were found in the 2 kb upstream region among five developmental stages. Interestingly, the CG methylation level of the TE-body region was less at T2 than that at other stages (Figure 2C). The CHH methylation level of the TE-body and 2-kb downstream region was methylated the least at T1 (Figure 2C). Circos plots of DNA methylation at later stages compared with T1 uncovered the distinctions in the three contexts at different genomic regions (Figure 3 and Supplementary Figure 4).

### Identification, Distribution, and Functional Annotation of DMRs and DMPs During Grain Filling in Foxtail Millet

**FIGURE 4:**
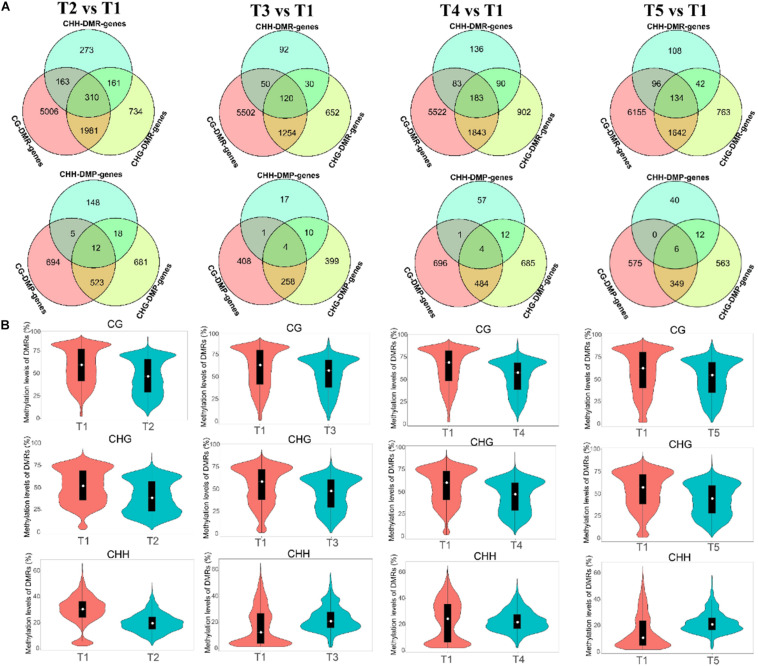
Differentially methylated regions (DMRs) and DNA methylation levels at the T2 vs. T1, T3 vs. T1, T4 vs. T1, and T5 vs. T1 comparisons. **(A)** Venn analysis of genes with DMRs in the CG, CHG, and CHH contexts of gene-body regions (DMR genes) and promoter regions [differentially methylated promoter (DMP) genes] among the different comparisons. **(B)** DMR methylation levels in the CG, CHG, and CHH contexts of different comparisons.

The differentially methylated region was analyzed for understanding the methylation differences at later stages compared with T1. The number of hyper-DMRs identified at later stages relative to T1 was gradually increased from T2 to T3, then decreased from T3 to T4, and increased again from T4 to T5. The hypo-DMRs showed an opposite trend. Furthermore, the number of CG DMRs was far more than other types of DMRs (Supplementary Figure 5). In CG and CHG context, the exon and intergenic regions had the most DMRs, respectively. In the CHH context, the promoter regions had the most hypo-DMRs, while hyper-DMRs were mostly distributed in the intergenic (T2 and T4), exon (T3), and promoter (T5) (Supplementary Figure 6). The numbers of genes with DMRs in gene-body regions (DMR_genes) and promoter regions (DMP_genes) among the different comparisons were shown in Venn analysis. For example, a total of 7,460, 2,186, and 907 DMR genes were identified for CG, CHG, and CHH contexts in the T2 vs. T1, and the number of DMP genes was 1,234, 1,234, and 183 (Figure 4A). Additionally, the methylation levels of the DMRs were decreased at later stages, but T3 and T5 showed higher CHH methylation levels than those in the T1 stage (Figure 4B).

### DNA Methylation Is Regulated by DNA Methyltransferase and Demethylase

A total of 11 methyltransferases, including 3 chromomethylases (CMT), 4 domain-rearranged methyltransferases (DRM), 3 methyltransferase 1 (MET1), and 1 DNA methyltransferase-2 (DNMT2), were identified (Supplementary Table 3). Among the 11 methyltransferase genes, *CMT3*, *DRM3*, *MET1-1*, and *MET1-3* are barely expressed (FPKM < 1) at all five stages (Supplementary Table 3). The foxtail millet genome harbors 7 DNA demethylase homologs, including 2 DEMETER (DME), 1 DEMETER-like2 (DML2), DEMETER-like3 (DML3), and 3 Repressor of Silencing1 (ROS1). *DML2* and *ROS1-3* are barely expressed (FPKM < 1) at each developmental stage (Supplementary Table 3).

A correlation analysis was performed to analyze the relationship among DNA methylation levels, DNA methyltransferase, and demethylase (Supplementary Table 4). The expression level of *SiCMT2* and *SiDRM4* were highly positively correlated with methylation levels of C, CG, and CHG, but showed a highly negative correlation with CHH. The expression level of *SiDRM2* was highly positively correlated with methylation levels of CG but showed a highly negative correlation with C, CHG, and CHH. The expression level of *SiMET1-2* was highly positively correlated with methylation levels of C and CHH.

The deoxyribonucleic acid demethylase gene *SiDME1* showed a highly positive correlation between expression and methylation level of C and CG. The expression level of *SiDME2* was highly positively correlated with methylation levels of C and CHG but showed a highly negative correlation with methylation levels of CHH. The expression levels of *SiDML3* and *SiROS1-1* were highly positively correlated with the methylation levels of CHH and C.

### Regulation of Transcriptional Dynamics by DNA Methylation

**FIGURE 5:**
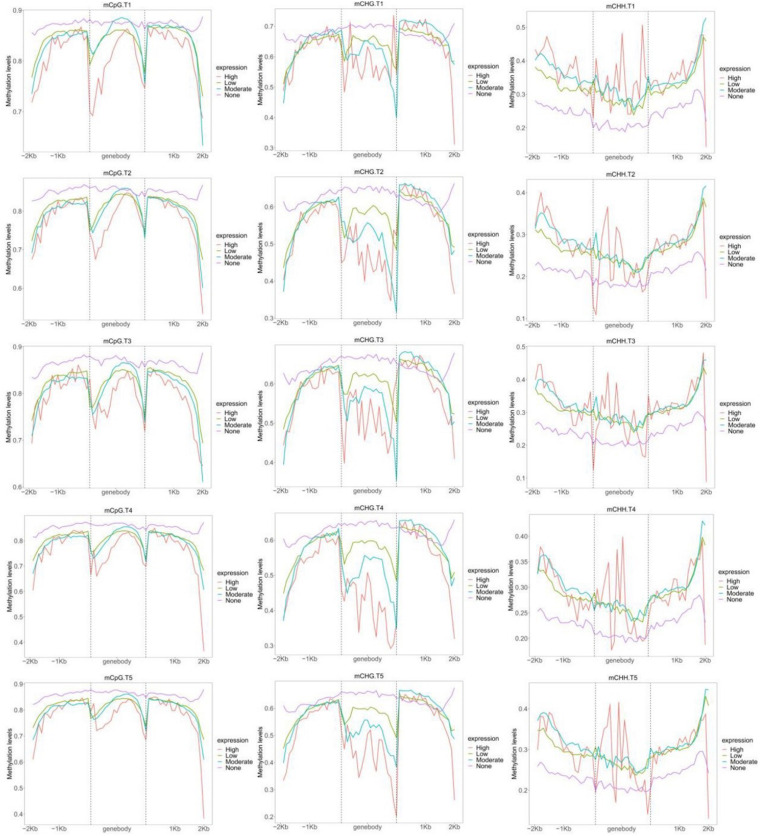
Distribution of CG, CHG, and CHH methylation levels within gene bodies based on four different expression levels: none, high, moderate, and low.

Based on the FPKM, all genes were classified into four groups (Figure 5). Genes in the none-group were highest CG methylated in all genetic regions (Figure 5). The levels of none-expression genes of CHG methylation were highest in gene-body and upstream. By contrast, genes in the none-group were the lowest CHH methylated in all genetic regions. As expected, high expression genes had the lowest CG and CHG methylation and the highest levels of CHH methylation in gene-body. In low and moderate groups, no significant correlation was found between gene expression and methylation status of CG and CHH in most genetic regions. By contrast, genes with higher expression levels presented lower CHG methylation levels in gene-body (Figure 5).

**FIGURE 6:**
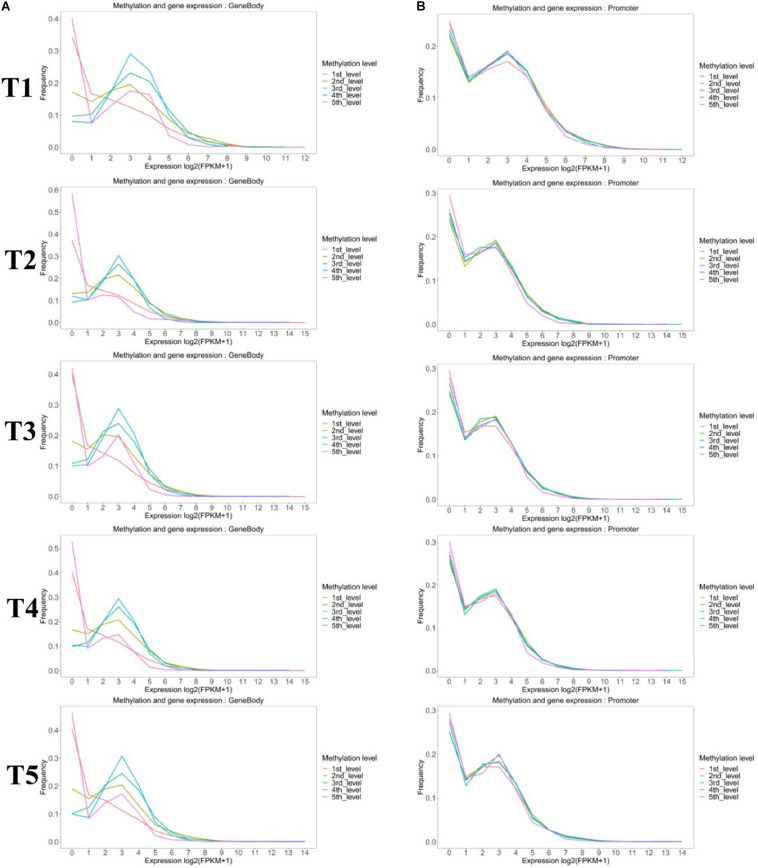
Comparison of the expression profiles of methylated and unmethylated genes. Methylated genes were further divided into quintiles based on **(A)** gene-body and **(B)** promoter methylation levels as follows: first: methylation level < 20%; second: 20% <methylation level <40%; third: 40% <methylation level <60%; fourth: 60% <methylation level <80%; and fifth: methylation level > 80%.

Methylated genes were also grouped by the promoter and gene-body methylation levels (Figure 6). The methylation level in the gene-body region was positively correlated with the gene expression level, except the high methylated genes (fifth group), which had the lower expression levels (Figure 6A). However, the methylation level in the promoter region of most genes had no significant effect on gene expression, except the genes in the first group that also showed a lower expression level (Figure 6B).

### Analysis of DEGs Associated With DNA Methylation at T2 Stage Compared With T1

**FIGURE 7:**
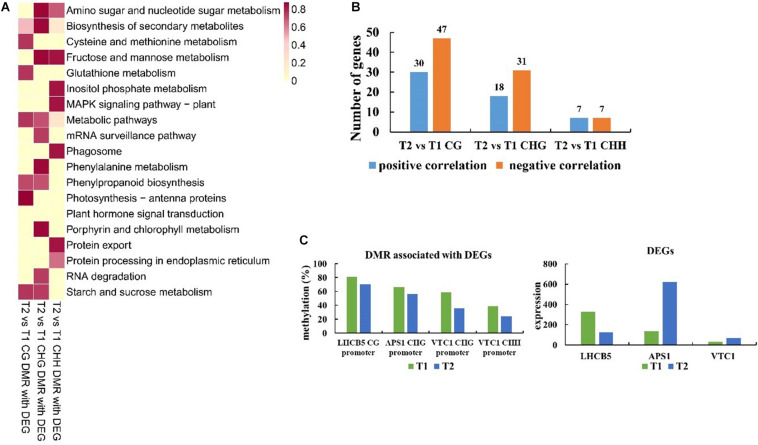
Function, methylation, and expression analysis of differentially expressed genes (DEGs) associated with CG, CHG, and CHH DMR for T2 vs. T1. **(A)** Kyoto Encyclopedia of Genes and Genomes (KEGG) analysis of DEGs associated with CG, CHG, and CHH DMR for T2 vs. T1. Enriched KEGG pathways are shown *via* heatmap. The scale represents –log_10_*^*p*^*^–value^ of enriched KEGG pathways. **(B)** The numbers of DEGs present a positive or negative correlation between methylation and expression levels. **(C)** Methylation and expression levels of specific genes between T2 and T1.

To verify the effect of altered DNA methylation on DEGs, we focused on the overlapped genes of DMGs and DEGs for different comparisons. The correlation between DEGs and methylation was calculated by Pearson correlation analysis based on the expression level of DEGs and methylation level of DMR. We identified the DEGs associated with DMR in line with Pearson correlation ≥ 0.90 (positive correlation) or ≤–0.90 (negative correlation). There were 77, 49, and 14 DEGs associated with CG, CHG, and CHH DMR for T2 vs. T1, respectively (Figure 7B). DEGs associated with CG DMR were significantly enriched in photosynthesis-antenna proteins, metabolic pathways, and cysteine and methionine metabolism, respectively (Figure 7A). Representative CG DMRs in the promoter of *LHCB5* (which encoded chlorophyll a-b binding protein CP26) were methylated less at T2 than at T1 and expressed at significantly lower levels at T2 than at T1.

The enriched categories of DEGs associated with CHG DMR included amino sugar and nucleotide sugar metabolism, biosynthesis of secondary metabolites, and phenylalanine metabolism (Figure 7A). Representative DMRs were methylated less at T2 than that at T1, such as *APS1* (which encoded glucose-1-phosphate adenylyltransferase small subunit) and *VTC1* (which encoded mannose-1-phosphate guanylyltransferase 1) in the CHG context in the promoter, whereas an opposite trend was observed in the expression level (Figure 7C).

Differentially expressed genes associated with CHH DMR were enriched in fructose and mannose metabolism, inositol phosphate metabolism, and protein export. In addition, representative CHH DMRs of *VTC1* were methylated less at T2 than at T1, also expressed at significantly higher levels at T2 than at T1.

### Analysis of DEGs Associated With DNA Methylation at T3 Stage Compared With T1

For T3 vs. T1, 245, 137, and 24 DEGs associated with CG, CHG, and CHH DMR were identified, respectively (Supplementary Figure 7B). KEGG enrichment of DEGs associated with CG DMR showed that the four most significantly altered pathways were metabolic pathways, glyoxylate, and dicarboxylate metabolism, inositol phosphate metabolism, and starch and sucrose metabolism, respectively (Supplementary Figure 7A). Representative CG DMRs in promoter or gene-body of four DEGs associated with starch and sucrose metabolism, *SS4* (which encoded soluble starch synthase), *PHO* (which encoded alpha-1,4 glucan phosphorylase L isozyme), *DPE1* (which encoded 4-alpha-glucanotransferase), and *BGLU31* (which encoded beta-glucosidase 31) were methylated less at T3 than at T1 but expressed at significantly higher levels at T3 than at T1. In contrast, the expression level of *FRK6* (which encoded fructokinase-6) was positively correlated with its methylation (Supplementary Figure 7C).

Differentially expressed genes associated with CHG DMR were enriched in seleno compound metabolism, other glycan degradation, and biosynthesis of secondary metabolites, respectively (Supplementary Figure 7A). Representative CHG DMRs in the promoter of *MS1* (which encoded Cobalamin-independent methionine synthase 1) were methylated less at T3 than that at T1, and also expressed at significantly lower levels at T3 than at T1.

Differentially expressed genes associated with CHH DMR were enriched in porphyrin and chlorophyll metabolism, MAPK signaling pathway, and plant hormone signal transduction (Supplementary Figure 7A). The methylation of CHH DMR in gene-body of *CHLH* (which encoded magnesium-chelatase subunit) showed a negative correlation with its expression level.

### Analysis of DEGs Associated With DNA Methylation at T4 Stage Compared With T1

For T4 vs. T1, 150, 134, and 34 DEGs associated with CG, CHG, and CHH DMR were identified respectively (Supplementary Figure 8B). KEGG pathway analysis of DEGs associated with CG DMR indicated that the three most significantly changed pathways were pyrimidine metabolism, oxidative phosphorylation, and purine metabolism (Supplementary Figure 8A). Representative CG DMRs in gene-body of four DEGs associated with pyrimidine metabolism, *POLA* (DNA polymerase I A), *pyrH* (uridylate kinase), *RNR1* (ribonucleoside-diphosphate reductase large subunit), and *PBY1* (5′-nucleotidase SurE) were methylated less at T4 than at T1, and the majority of them were expressed at significantly lower levels at T4 than at T1 except for *POLA* (Supplementary Figure 8C).

Kyoto Encyclopedia of Genes and Genomes pathway analysis demonstrated that the DEGs associated with CHG DMR were primarily related to photosynthesis, beta-alanine metabolism and ascorbate, and aldarate metabolism (Supplementary Figure 8A). Representative CHG DMRs in promoter and gene-body of *FDX5* (which encoded ferredoxin) and *FDC1* (which encoded ferredoxin-2) were methylated less at T4 than at T1 and were expressed at significantly lower levels at T4 than at T1.

Differentially expressed genes associated with CHH DMR were enriched in the biosynthesis of unsaturated fatty acids, alpha-linolenic acid metabolism, and pentose phosphate pathway (Supplementary Figure 8A). The methylation of CHH DMR in the promoter of *ACX4* (which encoded acyl-coenzyme A oxidase 4) showed a positive correlation with its expression level.

### Analysis of DEGs Associated With DNA Methylation at T5 Stage Compared With T1

There were 249, 147, and 28 DEGs associated with CG, CHG, and CHH DMR identified at T5 compared with T1, respectively (Supplementary Figure 9B). The top three KEGG pathways of DEGs associated with CG DMR were nitrogen metabolism, base excision repair, and anthocyanin biosynthesis (Supplementary Figure 9A). Representative CG DMRs in promoter or gene-body of DEGs associated with nitrogen metabolism, *ACA7* (alpha carbonic anhydrase 7) and *GLN2* (glutamine synthetase) were methylated and expressed less at T5 than that at T1, but the expression level of *NRT2*.*4* (high-affinity nitrate transporter 2.4) was negatively correlated with its methylation.

The functional enrichment of DEGs associated with CHG DMR primarily included arginine and proline metabolism, phenylalanine, tyrosine, and tryptophan biosynthesis, and tropane, piperidine, and pyridine alkaloid biosynthesis (Supplementary Figure 9A). Representative CHG DMRs in the promoter of *PAO1* (which encoded polyamine oxidase 5) and *ASP5* (which encoded aspartate aminotransferase) were methylated less at T5 than that at T1 and expressed at significantly lower levels at T5 than at T1.

Differentially expressed genes associated with CHH DMR were enriched in the pentose phosphate pathway, fructose, and mannose metabolism, and MAPK signaling pathway (Supplementary Figure 9A). The methylation levels of CHH DMR in gene-body and promoter of *PFP-ALPHA* (which encoded pyrophosphate–fructose 6-phosphate 1-phosphotransferase subunit alpha) and *G6PDH* (glucose-6-phosphate 1-dehydrogenase) were higher at T5 than at T1 but expressed at significantly lower levels at T5 than at T1.

## Discussion

Deoxyribonucleic acid methylation mediates plant development and phase transition. In apple, DNA methylation influenced apple flower bud formation (Xing et al., 2019). During the juvenile-to-adult phase transition, there was a significant correlation between DNA methylation and gene expression in *Malus hupehensis* (Xing et al., 2020). The genomic DNA methylation level was gradually increased during the ripening stage of sweet orange (Huang et al., 2019). DNA methylation dynamic played a pivotal role during seed development in chickpeas (Rajkumar et al., 2020). Nonetheless, the potential effect of epigenetic regulation on grain filling remains unknown despite the works done regarding its grain development and filling. Integrated herein is the epigenome and transcriptome analysis to gain new insights into DNA methylation in foxtail millet.

The contexts mCG were dominated followed by mCHG and mCHH in *Arabidopsis thaliana* and *mulberry* (Lister et al., 2008; Li et al., 2020). However, mCHH accounted for the highest proportion of total mC in the study, followed by the CG and CHG context. These findings are consistent with studies in apple trees, *M*. *hupehensis*, and cotton (Xu et al., 2018; Xing et al., 2019, 2020; Zhang et al., 2020). It is worth noting that CHH methylation remained elevated during grain filling (38–46%) and CG and CHG methylation showed the opposite trend (33–29 and 29–25%, respectively). Additionally, the fractional methylation levels in foxtail millet were similar with other plants, such as rice, *Beta vulgaris*, soybean, and poplar, with the highest levels in the CG context, followed by the CHG and CHH contexts (Li et al., 2008; Niederhuth et al., 2016). Previous studies have shown that DNA was methylated differently at different genetic regions, which also could affect gene expression (Ibarra et al., 2012; Liu et al., 2015). More methylated DNAs were found upstream of a gene in certain plants (Xing et al., 2019, 2020). However, in the present study, the CG methylation of gene-body was significantly higher than that in other regions, which was consistent with earlier reports in strawberry, orange, and peach (Cheng et al., 2018; Huang et al., 2019; Zhu et al., 2020).

Deoxyribonucleic acid methylation levels could be affected by the interaction, either coordination or antagonism, between DNA methyltransferase and demethylase. The regulation patterns varied from different plants between DNA methyltransferase and demethylase. During the fruit development, the expression of demethylase and DNA methylation level showed an opposite trend in orange and tomato (Huang et al., 2019; Wang et al., 2020a). The expression of DNA methyltransferase and demethylase could only affect methylation levels in specific contexts in some plants. For instance, only the methylation levels of C and CHH could be positively regulated by the expression level of *PpDRM1* in peach (Zhu et al., 2020). Sometimes, antagonism between DNA methyltransferase and demethylase affects the dynamic DNA methylation changes. For instance, drought induces DNA methylation levels in rice, but gene expression of methyltransferase and demethylase were both increased (Wang et al., 2020a). In this study, it is shown that the expression of some DNA methyltransferases and demethylase could enhance DNA methylation in specific contexts, and inhibit the methylation levels in others. Thus, the antagonism and synergy of DNA methyltransferases and demethylase could affect the dynamic change of methylation in specific contexts during grain filling in foxtail millet.

Deoxyribonucleic acid methylation-mediated gene regulation varied among different genetic regions. Global methylation and transcriptional analyses found that gene-body methylation could repress gene expression. DNA methylation in promoters may increase gene expression during flower bud formation in apples (Xing et al., 2019). In contrast, methylation analysis found that genes with unmethylated promoters presented a higher expression level and gene-body methylation may increase gene expression level under water deficit stress in apples (Xu et al., 2018). In cotton, genes with promoter DNA methylation had a low expression level, whereas the expression level of genes with gene-body methylation was higher than those without gene-body methylation (Zhang et al., 2020). Genes herein with hypermethylation in gene-body also showed a higher gene expression except for the highest methylated gene (fifth level). No distinct correlation was found between gene expression and methylated level in the promoters of most genes.

The specific role of methylated DNA in foxtail millet is largely unknown. To understand whether methylated DNA participated in transcription dynamic changes and the regulation of grain development, DEGs associated with DMR at T2, T3, T4, and T5 compared with T1 were identified. Previous studies demonstrated that the photosynthetic capacity and grain chlorophyll contents were closely related to grain filling rate in rice (Chen et al., 2020). In this study, DEGs involved in photosynthesis-antenna proteins, porphyrin and chlorophyll metabolism, and photosynthesis were identified as associated with CG, CHH, and CHG DMR, such as *LHCB5*, *CHLH*, and *FDX5*. Genes related to carbohydrate metabolism, nucleic acid, and protein metabolism were enriched for ontologies during grain development (Yu et al., 2016; Yang et al., 2017; Brinton and Uauy, 2019). As expected, DNA methylation altered the expression level of DEGs related to carbohydrate metabolisms, such as *APS1*, *VTC1*, and *MS1*, which were responsible for amino sugar and nucleotide sugar metabolism, fructose and mannose metabolism, and other glycan degradation, respectively. In addition, DEGs involved in cysteine and methionine metabolism and arginine and proline metabolism were also associated with DMR. It is well known that starch and sucrose metabolism were important factors for grain filling (Wang et al., 2015). In the current study, four DEGs associated with starch and sucrose metabolism, which are *SS4*, *PHO*, *DPE1*, and *BGLU31*, presented a negative correlation between methylation and their expression, but *FRK6* showed a positive correlation between its methylation and its expression level at T3 compared with T1. The results of this study demonstrated that the dynamic change of DNA methylation plays a crucial function in gene regulation, revealing the potential function of epigenetics in grain development in foxtail millet. This can be further studied using the currently more advanced genome editing technology, such as CRISPR/Cas, which studies gene function not only genetically but also epigenetically (Li et al., 2021; Zhang et al., 2021).

## Conclusion

In conclusion, we showed global DNA methylation dynamics and its regulatory function in gene expression in foxtail millet. Gene expression was negatively associated with CG and CHG DNA methylation, while that in the CHH context was positively associated with methylation in gene-body regions. The evaluation of the interconnection of the DNA methylome and transcriptome identified some stage-specific DEGs associated with grain filling, indicating that the expression of certain genes, involved in specific pathways, could be regulated by DNA methylation modification during grain development. We found, herein, that DNA methylation of different genetic regions has an important influence on the transcriptome changes, suggesting an epigenetic regulatory mechanism in the grain filling process in foxtail millet.

## Data Availability Statement

The datasets presented in this study can be found in online repositories. The names of the repository/repositories and accession number(s) can be found below: NCBI database with BioProject accession: PRJNA699635; BioSample: SAMN17804127 (https://www.ncbi.nlm.nih.gov/bioproject/PRJNA699635).

## Author Contributions

TW and RP conceived and designed the experiments. TW, QL, and HS performed the experiments. NH, PL, YW, YL, ZZ, and JL contributed reagents, materials, and analysis tools. TW, BZ, and RP wrote and revised the manuscript. All authors contributed to the article and approved the submitted version.

## Conflict of Interest

The authors declare that the research was conducted in the absence of any commercial or financial relationships that could be construed as a potential conflict of interest.

## Publisher’s Note

All claims expressed in this article are solely those of the authors and do not necessarily represent those of their affiliated organizations, or those of the publisher, the editors and the reviewers. Any product that may be evaluated in this article, or claim that may be made by its manufacturer, is not guaranteed or endorsed by the publisher.

## Footnotes

1
http://www.genome.jp/kegg/pathway.html
