# Supplementary material for: DNA Methylation and RNA-Sequencing Analysis Show Epigenetic Function During Grain Filling in Foxtail Millet (Setaria italica L.)
Source: Front Plant Sci. 2021 Aug 27;12:741415. doi: 10.3389/fpls.2021.741415 (PMC8429616; doi:10.3389/fpls.2021.741415)
Supplement: Supplementary file 1 [file Data_Sheet_1.PDF]

Supplementary materials:

Table S1 Details regarding the BS reads for the all samples

Samples	Raw reads	Clean reads	Uniqmap Reads	Mapping Rate	Convert Rate	GC Content	>Q20	>Q30
T1_1	135725864	131249912	96279142	83.48	99.61014585	28.77%	96.73%	92.43%
T1_2	152617086	148102336	110511970	83.46	99.60597285	28.18%	96.63%	92.29%
T1_3	126390712	122107374	89348404	82.05	99.60934782	28.09%	96.27%	91.55%
T2_1	144141152	139678214	102595020	81.58	99.60037642	27.71%	96.53%	92.10%
T2_2	137534596	133403976	96972506	80.78	99.5641802	27.28%	96.52%	92.10%
T2_3	164569614	159267558	117052964	82.3	99.60085021	28.07%	96.68%	92.38%
T3_1	116060878	112729776	82714032	82.2	99.61188322	28.49%	96.68%	92.33%
T3_2	137547874	133529216	95154324	80.3	99.63083164	28.52%	96.80%	92.61%
T3_3	122786810	119316182	87451338	82.01	99.58585209	28.19%	96.70%	92.40%
T4_1	171806786	166798922	118250730	79.32	99.5908547	28.32%	96.66%	92.31%
T4_2	132417854	128687858	90015406	77.85	99.63289981	28.22%	96.50%	92.00%
T4_3	125186868	121640684	87859926	80.16	99.59830939	27.74%	96.61%	92.27%
T5_1	144774360	140554060	100870196	79.6	99.57656867	27.66%	96.43%	91.87%
T5_2	186930950	182087056	124940978	76.6	99.62594407	28.29%	96.71%	92.46%
T5_3	162637614	157894626	111903036	79.16	99.59994353	27.84%	96.54%	92.10%

Table S2 Statistic of sequencing depth and covering of samples at five different stages

Sample	Site number	Mean coverage	Coverage (1X)	Coverage (5X)	Coverage (10X)
T1_1	381150210	34.27	93.91	90.66	86.39
T1_2	382841016	39.97	94.33	92.16	90.31
T1_3	381784099	32.38	94.07	90.97	86.62
T2_1	382671185	37.10	94.28	92.04	90.14
T2_2	382398268	35.31	94.22	91.43	87.81
T2_3	383229866	42.24	94.42	92.26	90.42
T3_1	381444635	30.02	93.98	91.09	87.21
T3_2	382055585	34.16	94.13	91.77	89.45
T3_3	381443357	31.81	93.98	90.65	86.12
T4_1	382906103	43.05	94.34	91.92	89.10
T4_2	382111425	32.76	94.15	91.75	89.50
T4_3	382134594	32.14	94.15	91.27	87.44
T5_1	384341046	37.04	94.70	91.92	88.22
T5_2	383223462	45.45	94.42	92.36	90.62
T5_3	381771043	40.91	94.06	90.51	86.36

Note: (1) Site number: the number of genomic sites in each sample; (2) Mean coverag: Average coverage depth of all genomic sites; (3) Coverage1X/5X/10X: the proportion of bases greater than or equal to 1/5/10 X sequencing depth in the genome to the total length of the genome.

Table S3 The expression levels of methyltransferase and demethylase related genes in foxtail millet during grain filling

Gene_ID	Gene_Symbol	T1	T2	T3	T4	T5
LOC101755233	*SiCMT1*	2.22	4.02	5.63	8.29	4.49
LOC101774591	*SiCMT2*	11.99	9.60	4.56	2.81	3.16
LOC101776623	*SiCMT3*	0.24	0.67	0.30	0.17	0.15
LOC101761707	*SiDRM1*	7.80	6.62	6.57	6.60	5.89
LOC101781283	*SiDRM2*	5.18	4.39	4.68	3.83	1.44
LOC101786351	*SiDRM3*	0.04	0.31	0.70	0.84	0.72
LOC101784586	*SiDRM4*	17.90	15.09	14.69	15.52	12.42
LOC101780757	*SiMET1-1*	0.02	0.04	0.05	0.08	0.02
LOC101778313	*SiMET1-2*	13.79	22.92	19.11	11.75	9.83
LOC101757301	*SiMET1-3*	0.27	0.35	0.11	0.05	0.04
LOC101758674	*SiDNMT2*	9.41	7.14	7.68	9.66	9.67
LOC101760859	*SiDME1*	14.02	8.40	9.35	8.20	6.59
LOC101769541	*SiDME2*	8.43	5.79	6.09	6.24	5.48
LOC101778802	*SiDML2*	0.16	0.24	0.38	0.66	0.47
LOC101785504	*SiDML3*	2.22	1.97	1.47	1.23	1.50
LOC101778625	*SiROS1-2*	6.86	4.81	7.28	8.56	6.55
LOC101762067	*SiROS1-1*	8.33	8.34	13.47	10.90	10.16
LOC111255678	*SiROS1-3*	0.19	0.29	0.41	0.61	0.78

Table S4 Correlation between transcript levels of DNA methyltransferase and demethylase genes and methylation levels of C, CG, CHG and CHH.

Gene_ID	Gene_Symbol	C	CG	CHG	CHH
LOC101755233	*SiCMT1*	-0.34	-0.50	0.13	0.16
LOC101774591	*SiCMT2*	0.72	0.58	0.52	-0.56
LOC101761707	*SiDRM1*	0.21	0.06	0.16	-0.13
LOC101781283	*SiDRM2*	-0.67	0.84	-0.94	-0.58
LOC101784586	*SiDRM4*	0.72	0.62	0.66	-0.83
LOC101778313	*SiMET1-2*	0.72	-0.08	0.19	0.73
LOC101758674	*SiDNMT2*	0.08	0.50	0.43	0.02
LOC101760859	*SiDME1*	0.96	0.73	0.09	-0.33
LOC101769541	*SiDME2*	0.96	0.13	0.63	-0.75
LOC101785504	*SiDML3*	0.16	0.02	0.42	0.58
LOC101762067	*SiROS1-1*	0.62	0.11	-0.34	-0.05
LOC101778625	*SiROS1-2*	0.28	0.04	0.29	0.17

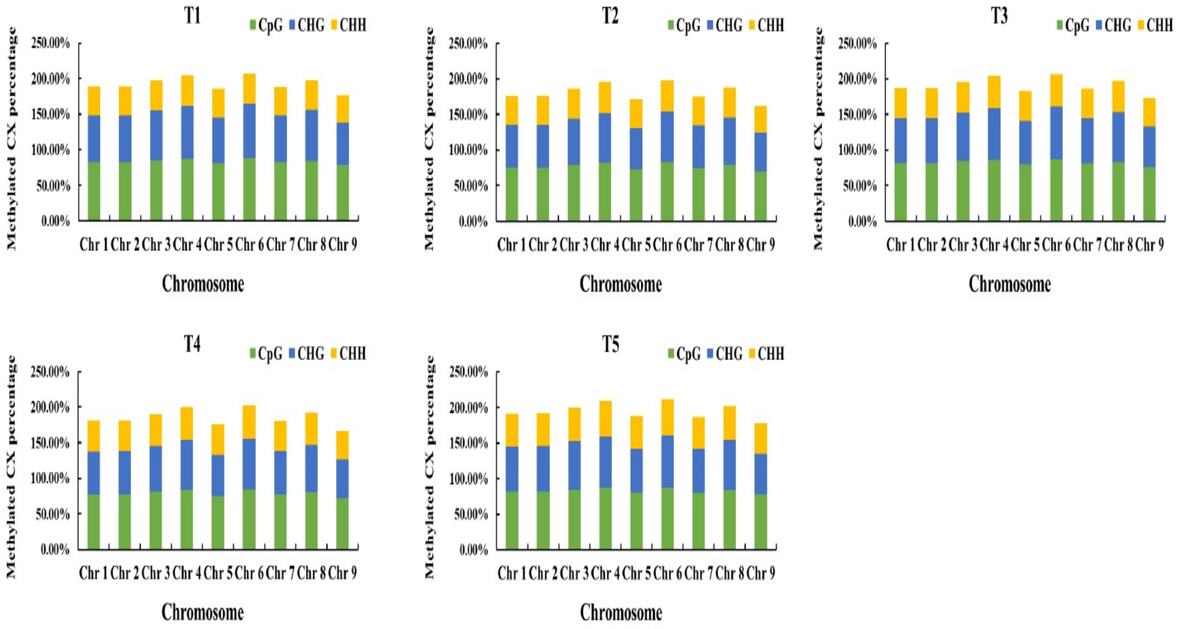

Figure S1: Percentage of 5mC methylation in the CG, CHG, and CHH contexts of each chromosome.

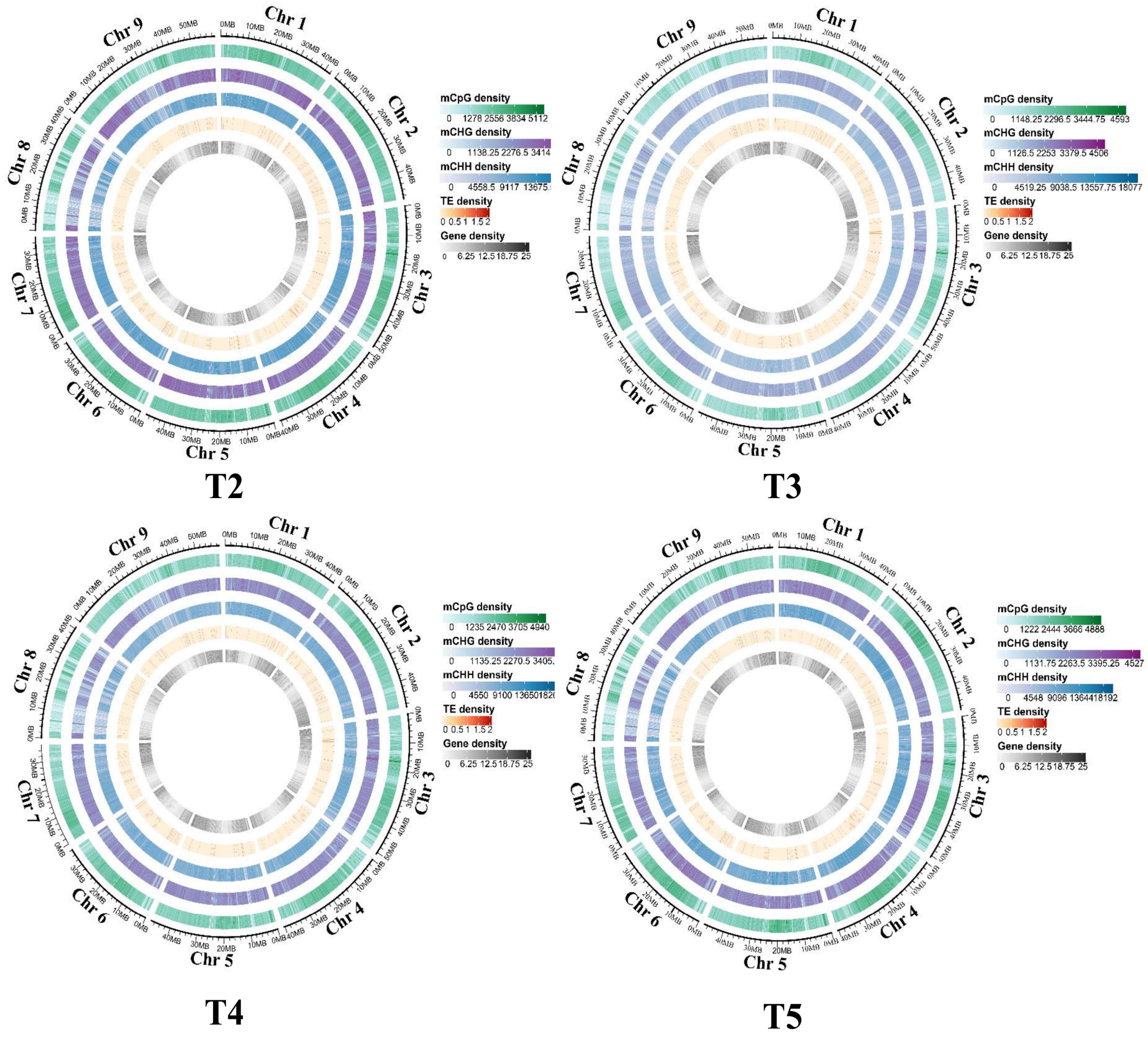

Figure S2: Circos plots of foxtail millet chromosomes. Track order: density plot of 5mC in CG, CHG and CHH contexts; density of TEs; gene density of each chromosome at the T2, T3, T4 and T5.

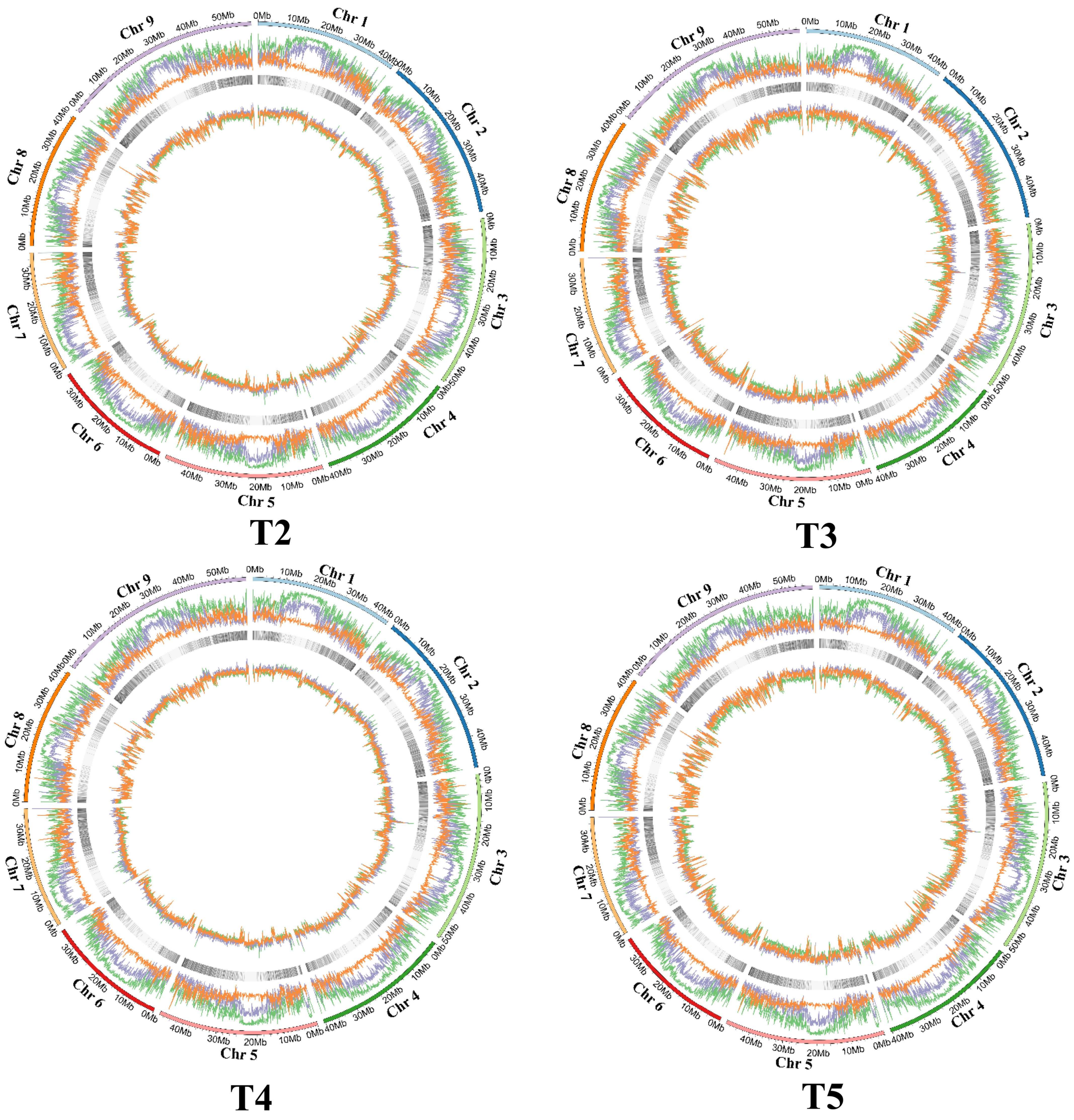

Figure S3: Density plot of 5mC in CG (green), CHG (purple) and CHH (orange) contexts in the gene bodies on each chromosome at the T2, T3, T4 and T5.

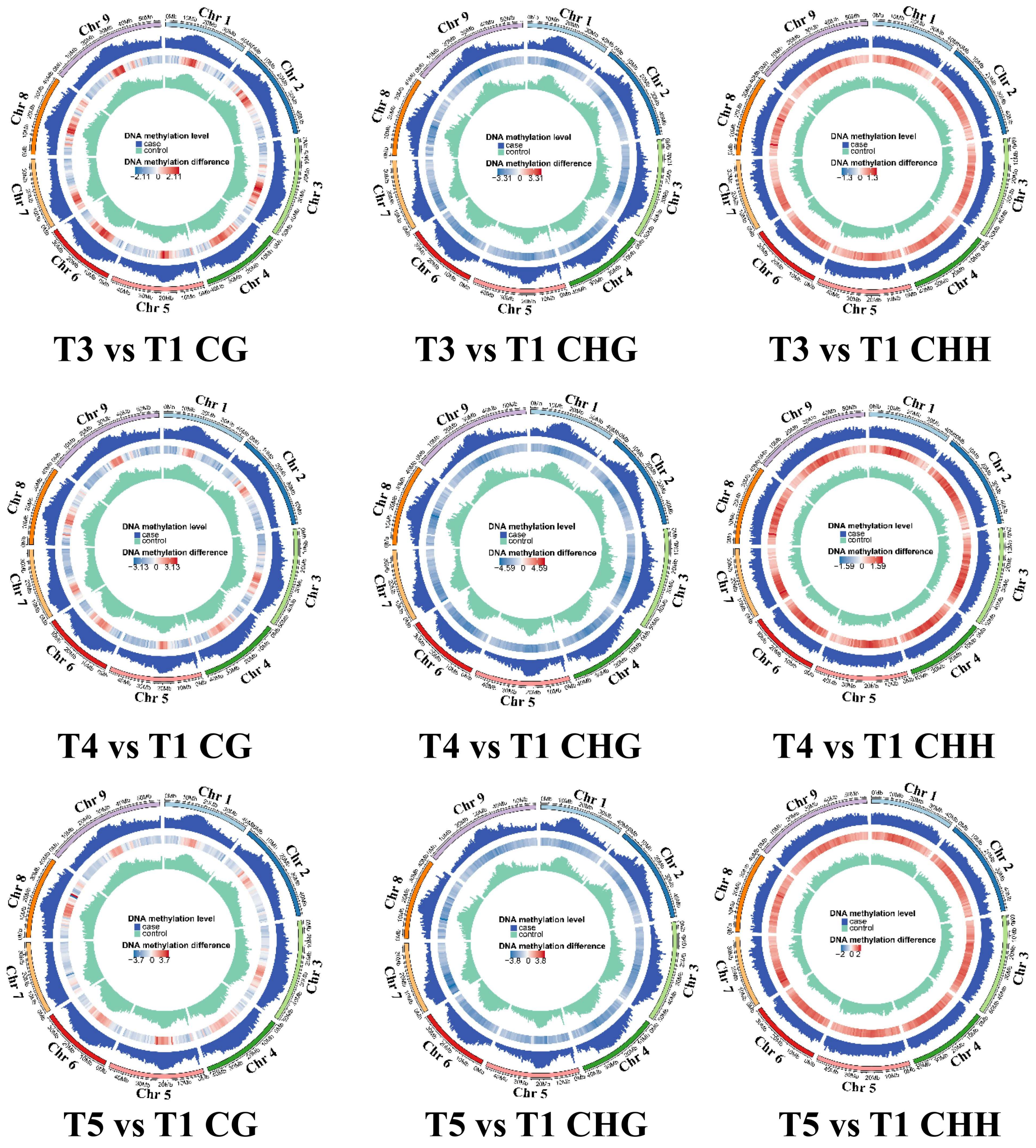

Figure S4: Comparative analysis of DNA methylation levels in different genomic regions.

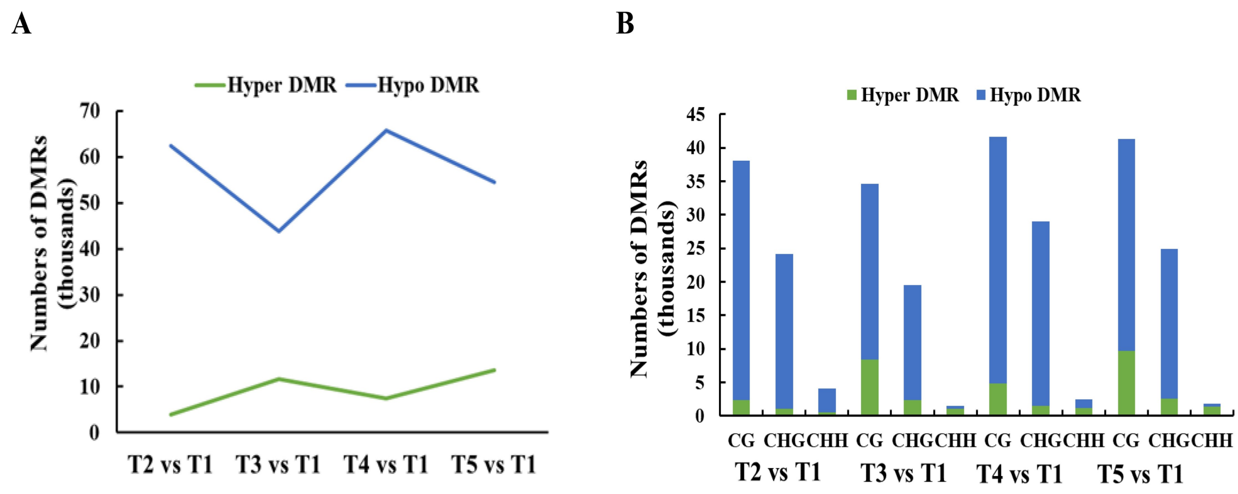

Figure S5: Number of DMRs, including hyper/hypomethylated DMRs in different contexts.

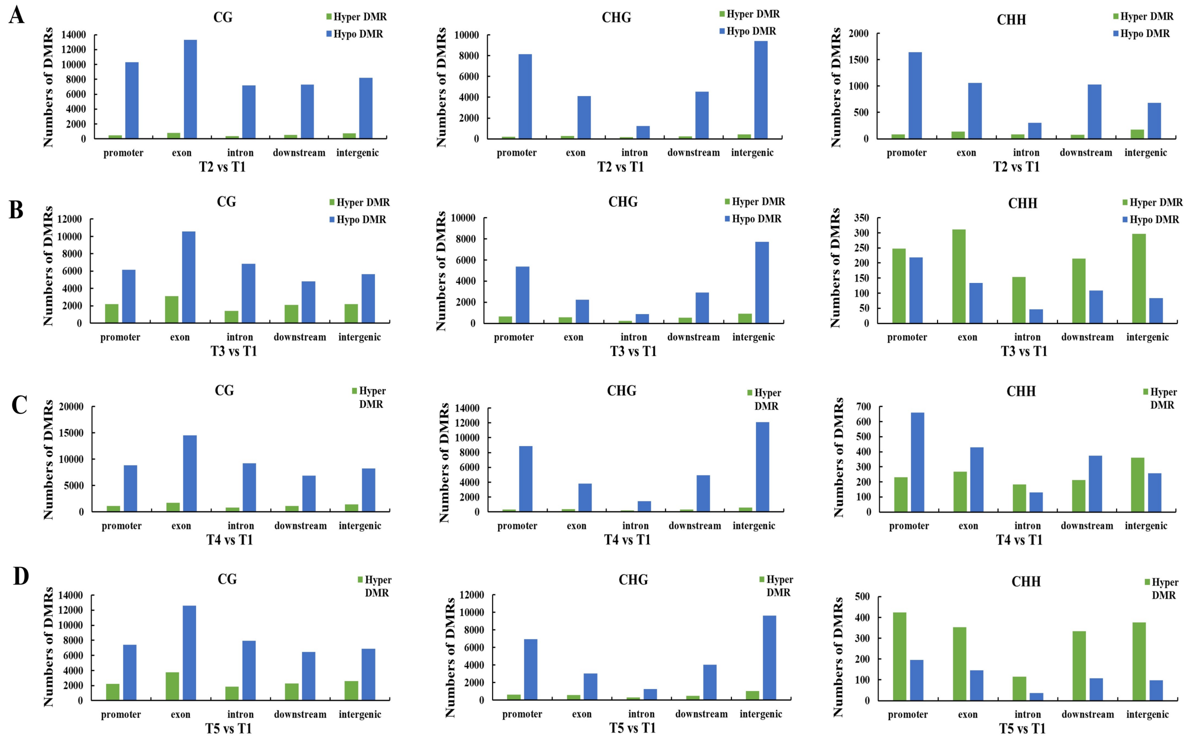

Figure S6: Number of DMRs, including hyper/hypomethylated sequences in the promoter, exon, intron, downstream and intergenic, among different comparisons.

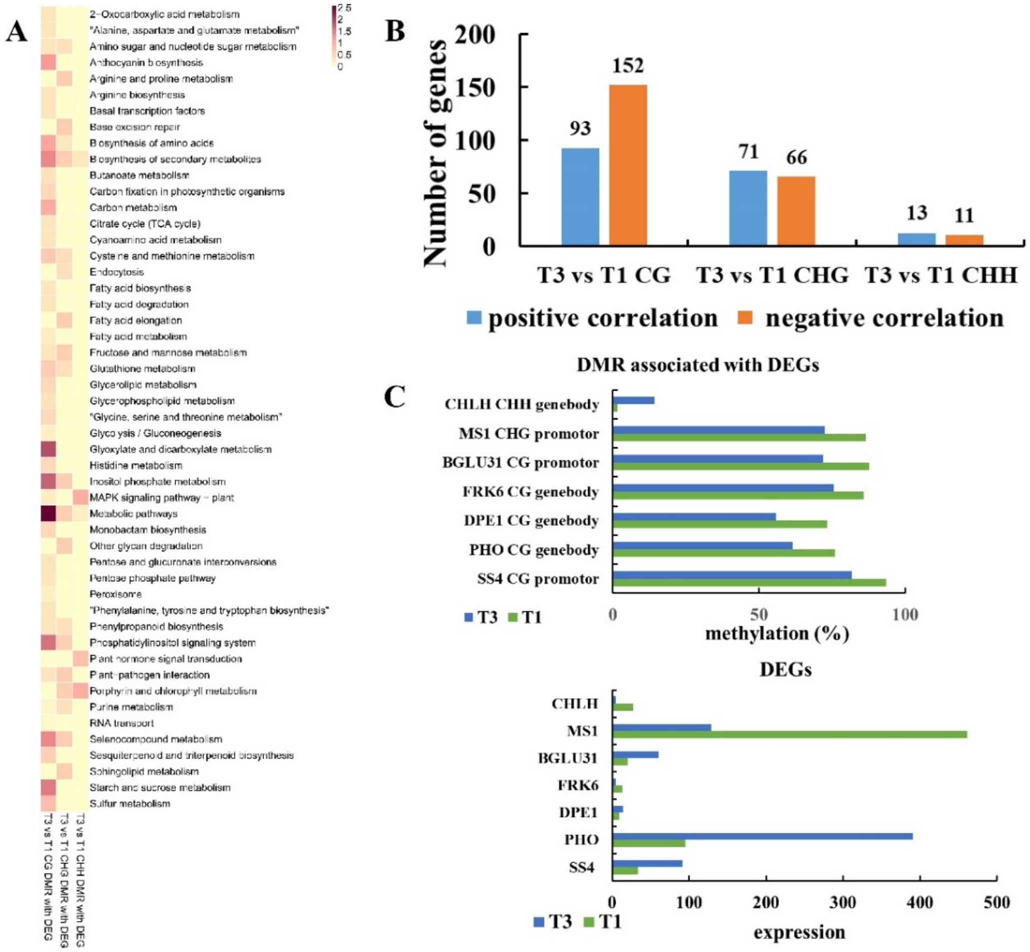

Figure S7. Function, methylation and expression analysis of DEGs associated with CG, CHG and CHH DMR for T3 vs T1. (A) KEGG analysis of DEGs associated with CG, CHG and CHH DMR for T3 vs T1. Enriched KEGG pathways are shown via heatmap. Scale represents $-\log_{10}{}^{p\text{-}value}$ of enriched KEGG pathways. (B) Numbers of DEGs which presented positive or negative correlation between methylation and expression levels. (C) Methylation and expression levels of specific genes between T3 and T1.

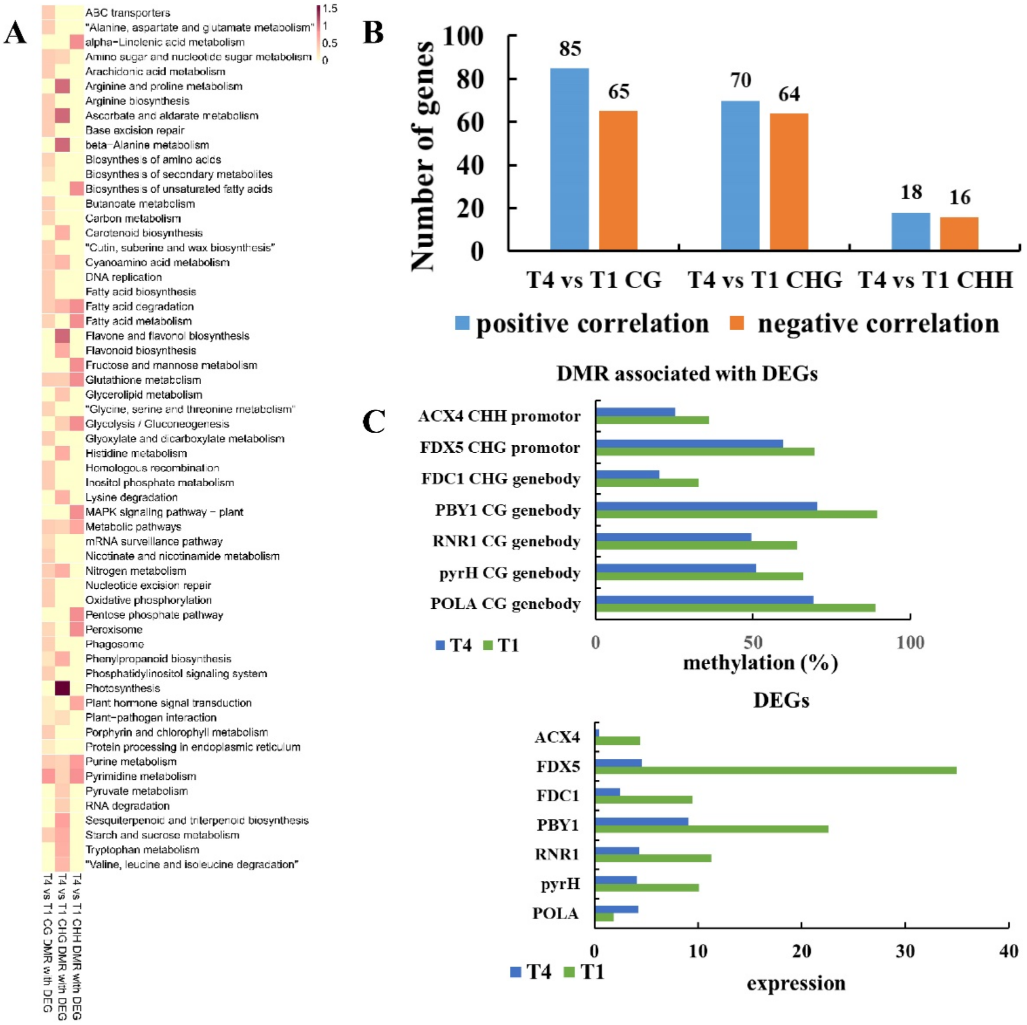

Figure S8. Function, methylation and expression analysis of DEGs associated with CG, CHG and CHH DMR for T4 vs T1. (A) KEGG analysis of DEGs associated with CG, CHG and CHH DMR for T4 vs T1. Enriched KEGG pathways are shown via heatmap. Scale represents -log$_{10}$$^{p\text{-}value}$ of enriched KEGG pathways. (B) Numbers of DEGs which presented positive or negative correlation between methylation and expression levels. (C) Methylation and expression levels of specific genes between T4 and T1.

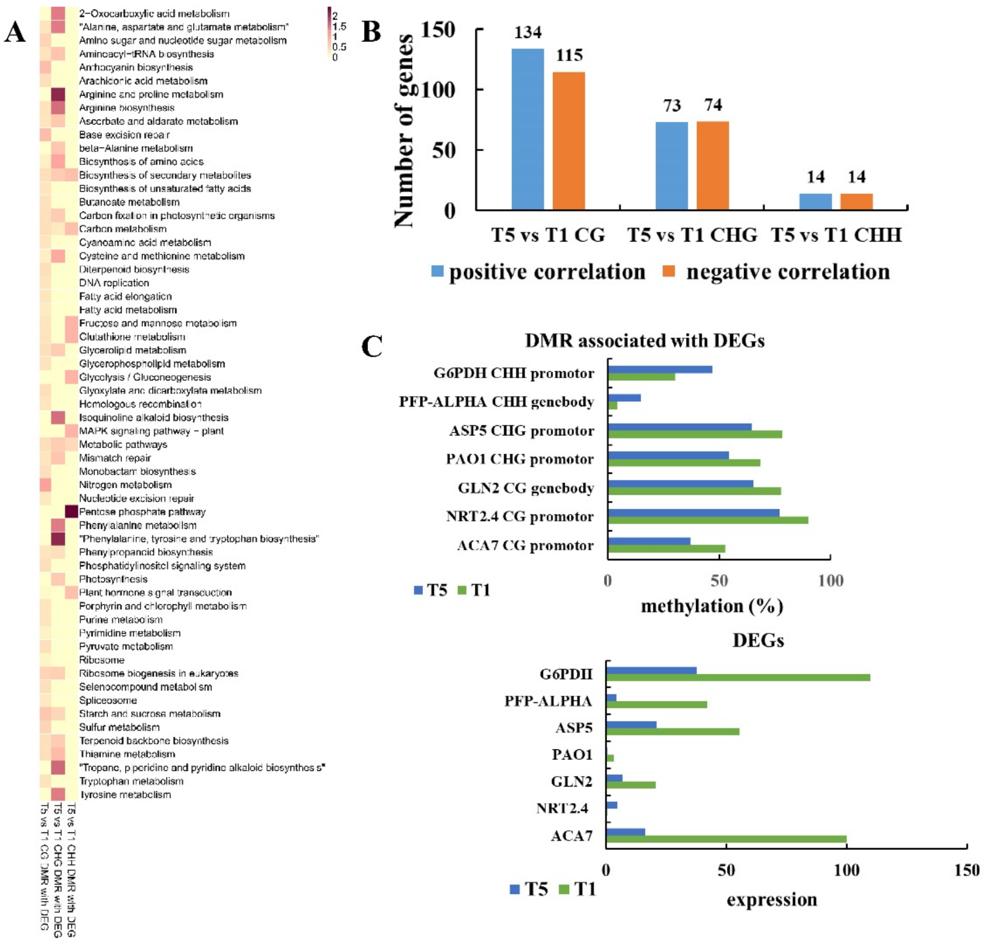

Figure S9. Function, methylation and expression analysis of DEGs associated with CG, CHG and CHH DMR for T5 vs T1. (A) KEGG analysis of DEGs associated with CG, CHG and CHH DMR for T5 vs T1. Enriched KEGG pathways are shown via heatmap. Scale represents $-\log_{10}{}^{\text{p-value}}$ of enriched KEGG pathways. (B) Numbers of DEGs which presented positive or negative correlation between methylation and expression levels. (C) Methylation and expression levels of specific genes between T5 and T1.
